# A Review of Thoracic Outlet Syndrome and the Possible Role of Botulinum Toxin in the Treatment of This Syndrome

**DOI:** 10.3390/toxins4111223

**Published:** 2012-11-07

**Authors:** Jacqueline Mary Foley, Heather Finlayson, Andrew Travlos

**Affiliations:** Department of Physical Medicine and Rehabilitation, University of British Columbia, GF Strong Rehabilitation Centre, 4255 Laurel Street, Vancouver V5Z 2G9, Canada; Email: heather.finlayson@vch.ca (H.F.); travlos@pacificrehab.net (A.T.)

**Keywords:** thoracic outlet syndrome, botulinum toxin

## Abstract

The objective of this paper is to discuss the classification, diagnosis, pathophysiology and management of Thoracic outlet syndrome (TOS). Thoracic outlet syndrome (TOS) is a complex entity that is characterized by different neurovascular signs and symptoms involving the upper limb. TOS is defined as upper extremity symptoms due to compression of the neurovascular bundle in the area of the neck just above the first rib. Compression is thought to occur at one or more of the three anatomical compartments: the interscalene triangle, the costoclavicular space and the retropectoralis minor spaces. The clinical presentation can include both neurogenic and vascular symptoms. TOS can be difficult to diagnose because there is no standardized objective test that can be used and the clinician must rely on history and several positive findings on physical exam. The medial antebrachial cutaneous nerve conduction may be a sensitive way to detect pathology in the lower trunks of the brachial plexus which is promising for future research. Treatment options continue to be conservative and surgical. However, for those who have failed physical therapy there is research to suggest that botulinum toxin may help with symptom relief. However, given that there has been conflicting evidence, further research is required using randomized controlled trials.

## 1. Introduction

Thoracic outlet syndrome (TOS) is a complex entity that is characterized by different neurovascular signs and symptoms involving the upper limb. A definition of TOS is “upper extremity symptoms due to compression of the neurovascular bundle in the area of the neck just above the first rib” [1]. Compression is thought to occur at one or more of the three anatomical compartments: the interscalene triangle, the costoclavicular space and the retropectoralis minor spaces [2]. The clinical presentation can include both neurogenic and vascular symptoms. There is a paucity of research on interventions for TOS to guide an evidence based approach to treatment. However, over the past decade there has been increasing interest in the role of botulinum toxin (BTX) in the management of musculoskeletal conditions including TOS.

The aim of this paper is to provide a review of the classification, diagnosis, pathophysiology and current management of TOS. It will then discuss the potential use of BTX in the treatment of TOS. The goals are to stimulate further discussion regarding the role of BTX in the treatment of TOS and to suggest areas of future study.

## 2. Classification

The classification of TOS can be based on etiology, symptoms, clinical presentation, or anatomy [2]. One proposed classification system that has received support breaks TOS into three anatomical categories. The neurovascular bundle consists of 3 structures: arteries, veins and nerves. TOS can be classified based on these structures, that is, arterial TOS, venous TOS and neurogenic TOS. It is widely believed that the vast majority of TOS is neurogenic, with this form comprising approximately 95% of all cases [2]. Neurogenic TOS (NTOS) involves compromise of the brachial plexus trunks or cords formed from nerves that come from the C5 to T1 spinal levels [3].

### 2.1. Arterial TOS

Arterial TOS (ATOS) makes up approximately 1% of cases and is the least common of the three types [1]. Patients typically present with ischemia of a digit, symptoms of claudication, paraesthesias, pain, pallor and decreased temperature in the hand. It is rare to have symptoms in the shoulder and neck [1]. The symptoms are secondary to subclavian artery stenosis or development of an aneurysm leading to thrombus formation with distal emboli. The symptoms usually do not develop until embolization has occurred. On physical examination the individual will have loss of peripheral pulse, notable colour change and ischemia of the digits [2]. This type of TOS is usually secondary to the presence of a cervical rib or anomalous first rib. Treatment of arterial TOS is surgical removal of the abnormal rib and reconstruction of the diseased segment of the subclavian artery [2,4].

### 2.2. Venous TOS

Venous TOS (VTOS) accounts for 2%–3% of cases [2]. It is a result of subclavian vein obstruction. This obstruction may be secondary to thrombosis, although a thrombus is not always present. It is thought that the thrombosis occurs because there has been an intrinsic narrowing of the subclavian vein caused by compression and scar tissue, which has formed as a result of repetitive compression injury to the subclavian vein between the clavicle and first rib [5]. The thrombosis formation is the secondary process. The progressive narrowing of the subclavian vein is at first asymptomatic since collateral vein expansion occurs simultaneously. At some point in time a thrombus develops within the obstructed subclavian vein, which can propagate peripherally with obstruction of the collateral vein origins. The key physical finding is swelling of the involved limb and possibly visible subcutaneous veins over the shoulder and upper chest [2]. The arm is discoloured, cyanotic and painful and the individual may experience paraesthesias. This is the acute clinical presentation of “effort thrombosis” [5]. Repetitive overhead work activities, or even swimming and throwing may act as a precipitant to the development of VTOS [2]. In the acute setting, the treatment is thrombolysis, surgical excision of the first rib with subclavian vein venolysis. Occasionally, it is necessary to perform dilatation of the residual stenosis by balloon angioplasty or surgical reconstruction with patch angioplasty or bypass. If it is a chronic presentation, with heaviness and an objectively swollen limb but the vein is still patent then decompression with rib resection, angioplasty or reconstruction is recommended [2,6].

### 2.3. Neurogenic TOS

Neurogenic TOS (NTOS) involves compression of the brachial plexus trunks or cords, comprised of nerves that come from the C5-T1 spinal levels [3]. The clinical picture is one of nerve irritation. Individuals with this syndrome often experience pain, paraesthesias and numbness in the neck, shoulder, arm and hand [3]. The paraesthesias are most often reported in all 5 fingers but worse in the fourth and fifth digits and medial forearm [2]. These symptoms are made worse by elevated, overhead, or outstretched positions of the arm [2,3,7,8]. Individuals will often have pain over the trapezius and the neck, occipital headaches and may even experience anterior chest wall pain [1,2]. 

On physical examination there may be tenderness over the scalene muscles and subcoracoid space. There is often decreased sensation to light touch in the fingers, especially over the fourth and fifth digits. A positive Tinel’s sign may be elicited over the area of the brachial plexus above the clavicle in the scalene triangle, with reproduction of paraesthesias in one or more of the nerve root distributions [9]. 

## 3. Provocative Clinical Tests

There are provocative tests that can reproduce symptoms of Neurogenic TOS by putting stress on the neurovascular bundle. These tests can be used to help with diagnosis. In order for the test to be considered positive it must elicit the patient’s symptoms. The brachial plexus tension test of Elvey has proven to be a useful clinical test [10]. This Brachial Plexus Tension Test or Upper Limb Tension Test uses maneuvers when used step wise increase tension within neural tissues related to the upper limb. It has been referred to as the upper limb equivalent of the straight leg raise of the leg. A clinical validation study of the BPTT found that the test had moderate to high intra-examiner reliability (0.825) and that it could be used in the clinical situation to discriminate between the presence or absence of brachial plexus involvement in patients with upper limb symptoms [11,12,13]. 

### 3.1. Elvey’s Test

Elvey’s Test involves the individual lying in supine. The shoulder joint is abducted and externally rotated. The examiner then adds shoulder girdle depression, forearm supination, wrist and finger extension and, finally, elbow extension. The position of lateral neck flexion to the contralateral side is used in the examination of the symptomatic arms if the other maneuvers are of full range and fail to provoke a symptomatic response [9]. This test has recently been modified and referred to as the modified Upper Limb Tension Test [1]. It involves having the patient in a seated position and performing the maneuvers actively, rather than passively. This allows both sides to be tested simultaneously and the unaffected arm can serve as a control for the affected limb. The patient abducts their shoulders to 90 degrees with the elbows in extension. The second position involves the patient actively dorsiflexing their wrists. The third position involves the patient tilting their head to their shoulder. Position one and two will elicit symptoms on the ipsilateral side. Position three will elicit symptoms on the contralateral side. A positive test is pain down the arm with reproduction of patient’s symptoms [1]. When a positive response is found it means that there has been compression of the nerve roots or branches of the brachial plexus in either the cervical spine, pectoralis minor space or the thoracic outlet [1].

### 3.2. Roos Test

Another test that has proven to have benefit in the diagnosis of NTOS is Roos Test. This test is sometimes referred to as the elevated arm stress test [1,9,14]. This test involves having the patient stand and abduct their arms to 90 degrees, elbows flexed at 90 degrees and slightly posterior to the frontal plane. The patient is then instructed to open and close their hand slowly for three minutes. If the patient is unable to keep their arms in this position for 3 min, experiences heaviness, profound weakness or reproduction of their symptoms the test is considered positive [11]. Others investigators describe this test as a one minute test [1,2,8].

### 3.3. Adson Test

Another common test for TOS is Adson Test [9]. In order for this test to be considered positive the examiner must find a decrease in pulse along with a reproduction of symptoms. Adson Test involves locating the patient’s radial pulse and the patient then rotates their head towards the test shoulder. The patient then extends their head while the examiner laterally rotates and extends the patient’s shoulder with the elbow in extension. The patient then takes a deep breath in and holds it [9]. The test is considered positive if there is loss of the radial pulse [2,15] and reproduction of symptoms [1,2]. 

Provocative tests that employ a vascular sign are not reliable in the diagnosis of a neurologic condition [1]. As such Adson Test and Roos Test should not be used to either rule in or rule out a NTOS [1]. Elvey’s Upper Limb Tension Test is most specific for neurologic pathology in the upper extremities and is considered a important component of the physical exam for NTOS [1,2].

## 4. Electrodiagnostic Studies

There is controversy regarding the use of electrodiagnostic studies for the diagnosis of TOS. Nerve conduction velocity (NCV) prolongation is seen in patients with long standing NTOS that results in atrophy of the muscle [2,16]. However, nerve conduction studies can be normal [2,16,17]. The ulnar sensory nerve action potential and compound motor action potential may be reduced and there may be abnormalities in F-wave latency in some individuals with NTOS [2,16]. There may be electromyography (EMG) results consistent with neurogenic damage, such as increased motor unit action potential amplitude and/or duration, and decreased recruitment at maximum effort. EMG may not be sensitive enough to pick up less severe NTOS [2,16,17,18]. Nerve conduction studies and EMG is useful clinically to rule out other neurologic disorders, such as a radiculopathy, carpal tunnel syndrome or motor neuron disease [2]. 

Recently, the medial antebrachial cutaneous (MAC) nerve conduction study has been identified as a sensitive test to detect milder NTOS. It measures the sensory function of the lower trunk of the brachial plexus. This test can be abnormal in those whose standard EMG/NCS are normal [2,19,20]. MAC studies may help to provide objective evidence of NTOS [2,19,20,21]. 

## 5. Vascular Studies

Arteriography and venography are not indicated for the diagnosis of NTOS [2]. Arteriography is necessary for surgical planning in ATOS only. Magnetic resonance angiography or computed tomographic angiography can be used to confirm arterial obstruction. In individuals who present with VTOS with arm swelling and cyanosis venography is the investigation of choice. Venography requires peripheral arm cannulation that can sometimes be challenging in the setting of significant peripheral edema. Magnetic resonance imaging (MRI), and computed tomography (CT), can be helpful for screening purposes and has the added advantage of imaging the surrounding anatomy. However, if a decision is made to progress to thrombolysis then successful cannulation of the peripheral vein is required through catheter based venography to establish patency of the vessel. Doppler ultrasound is sometimes used to assess for patency of the vessel, of these, color Doppler has a sensitivity of 70%–100% and a specificity of 93% [5]. However, ultrasound should not be used to exclude the diagnosis of thrombosis.

## 6. Pathophysiology of NTOS

Impingement of the neurovascular bundle supplying the upper limb can occur at different sites, but the interscalene triangle is frequently implicated [2,22,23]. This triangle is formed between the anterior and middle scalene muscles. Scalene muscle injury is emerging as the most common etiology of TOS. It is thought that this injury can be caused by a single significant traumatic event, or secondary to repetitive trauma over time. Overhead work activities may lead to this repetitive trauma, such that assembly line work, hair dressing, and cash register operation are jobs that are sometimes associated with this syndrome [23,24,25,26]. 

Research suggests that TOS most frequently occurs following a single episode of neck trauma such as a motor vehicle accident [26]. The individual typically experiences neck pain within days followed by symptoms in the hand and arm within weeks [2]. The trauma may cause scalene muscle hemorrhage resulting in edema in the region of the anterior and middle scalenes. This can eventually become fibrosed, scarred and result in spasm of the scalenes [2,3,23,27]. In fact, histologic studies of scalene muscles have demonstrated Type I fiber predominance along with Type II fiber pleomorphism and atrophy [2]. One study showed a significantly increased amount of connective tissue representing muscle scarring in the scalene in those with TOS compared to control subjects [22].

As scarring and spasm develop in the scalene, the muscles compress the brachial plexus. This compression leads to the symptoms of pain and paraesthesias in the upper extremity [6]. Those individuals with congenitally narrow thoracic outlet spaces, the presence of a cervical rib or cervical bands may be at greater risk of developing TOS following injury [2,3].

There are anatomic variations in the scalene triangle. The space between the anterior and middle scalenes can vary from very narrow to wide [2,3]. Sanders and Pearce [28] found that over 80% of individuals who had surgery for NTOS had a narrow interscalene space and the nerves tended to emerge higher up in the triangle and were touching the muscles as they emerged [28]. NTOS is sometimes associated with pectoralis minor pathology [29]. Those with pectoralis minor involvement will often have tenderness over the pectoralis minor tendon accompanying their symptoms of NTOS. Sanders reported success in treatment with a pectoralis minor tenotomy performed as an outpatient procedure under local anaesthesia with heavy sedation in select patients with pectoralis minor involvement [29]. Other researchers have postulated that anomalies in the subclavius muscle, often referred to as subclavious posticus, may play a role in the development of thoracic outlet syndrome [30,31,32]. This supernumerary muscle sometimes inserts on the superior surface of the sternal end of the first rib, runs laterodorsally and inserts on the superior margin of the scapula. These cadaveric studies suggest that this muscle may be implicated in TOS as this aberrant muscle runs on the anterior surface of the subclavian vein and crosses over the brachial plexus [32].

### 6.1. Cervical Ribs and Anomalous First Ribs

The incidence of cervical ribs and anomalous first ribs in the general population is 0.76% and 0.74% [2,29]. Seventy percent of cervical ribs are found in women and anomalous first ribs are equally divided between men and women [1,2,29]. The majority of cervical ribs and anomalous first ribs are asymptomatic. If an individual with a cervical rib or anomalous first rib experiences a neck injury this can predispose one to the develop TOS, the majority being neurogenic, and a minority arterial [2]. 

It is rare for patients with a cervical rib or anomalous first rib to spontaneously develop NTOS. Sanders reports that over a 28 year period and 1000 surgeries for NTOS there has been an incidence of less than 5% for cervical ribs and anomalous first ribs [2,27].

The pathophysiology of neurogenic TOS most often is a combination of neck trauma (including microtrauma secondary to repetitive activities) plus an anatomic predisposition that results in pathology of the scalene muscles and compression of the brachial plexus. This ultimately leads to the symptoms of NTOS.

## 7. Management of NTOS

The treatment of NTOS is often broken down into conservative or surgical treatment [2]. Conservative treatment focuses on physiotherapy involving stretching exercises for the neck and shoulder, with a focus on the scalene and pectoralis minor muscles. Postural training and ergonomic correction of a patient’s work station are important. Other modalities include heat and ultrasound to facilitate effective stretching of the scalene and pectoralis minor [2,33]. Gentle stretching, relaxation and biofeedback exercises are advocated [2,34,35]. Trigger point injections and medications such as anti-inflammatories and analgesia are used. NTOS can be made worse with strengthening exercises with heavy weights and neck traction [2]. 

A common treatment for TOS is scalenectomy with the goal of decompression of the interscalene space. This is either done alone or in combination with first rib resection [7,22]. Some surgeons advocate combining these two procedures to decrease the need for further surgeries. Some surgeons perform scalene muscle removal for suspected upper plexopathies and transaxillary first thoracic rib resection with suspected lower plexopathies [32]. Sanders [29] reported good outcomes with pectoralis minor tenotomy when there was evidence of pectoralis minor involvement on physical exam. The indications for surgery include a sound clinical diagnosis, disabling symptoms with loss of function and an insufficient response to conservative measures.

Non-surgical techniques to diminish pressure in the interscalene space by relaxing the scalene muscles are being explored in the treatment of TOS. These include anesthetic agents [7,36], steroids [7], and botulinum toxin type A (BTX-A) [3,37,38,39]. The use of scalene muscle injections with local anesthetics is very short-lived and as such will only provide adjunctive support to one’s clinical diagnosis and possible prognosis regarding the reversibility of symptoms. BTX-A injections have demonstrated an ability to result in more sustained improvement in symptoms [3,37,38,39,40]. 

## 8. Botulinum Toxins and Pain

BTX-A is a neurotoxin that is used to treat focal muscle hyperactivity, including muscle spasticity and dystonia [41]. The beneficial effect is the result of blockade of presynaptic nerve terminals releasing acyetylcholine [41,42,43,44]. When BTX-A was first being used there were anaecdotal findings that its use resulted in pain relief independent of its muscle relaxant properties [42]. Since that time, studies have suggested benefits of BTX-A in the treatment of chronic neuropathic pain [41,45,46] myofascial pain syndromes [47], chronic neck and low back pain [48] and even joint pain [49]. 

The antinociceptive effect of BTX-A is thought to be through one or more of three mechanisms: neuromuscular block at the level of the soluble *N*-ethylmaleimide sensitive factor attachment protein receptor, a protein complex within cholinergic nerve terminals and autonomic synapses; or inhibition of pain pathways by modulating the release of calcitonin gene related peptide and substance P; and/or an effect on the microvascular circulation. These factors lead to effects on pain that work both peripherally and centrally [50].

## 9. Anterior Scalene Muscle Injections

Non-surgical techniques to decrease compression in the interscalene triangle have included injections of anaesthetic agents [7], steroids [7] and BTX-A [3,37,38,39]. Jordan *et al.* [7] reported that anesthetic block of the anterior scalene muscle could be used to help predict which patients may potentially benefit from decompressive surgery for the treatment of TOS. They used electrophysiological (EMG) guidance to successfully demonstrate the location of the anterior scalene muscle in all 122 subjects. Using this technique they went on to inject an anaesthetic agent, and found that 90% of those with a clinical diagnosis of TOS had a positive response. Of 38 patients who went on to have surgical decompression 30 of 32 (94%) with a positive block had a good outcome compared with 3 of 6 (50%) who underwent surgery in spite of a negative block. The authors suggest that blocking the anterior scalene muscle under EMG guidance may help to predict which patients will benefit from surgical decompression of the thoracic outlet [7].

BTX-A has shown promise in its ability to provide more sustained symptom relief for patients with NTOS [3,37,38,39,40]. This gives it a significant advantage over temporary anaesthetic blockade [40]. Although, this may not be an appropriate comparison as anaesthetic blockade has been used primarily to enhance the assessment as opposed to treatment of NTOS. The rationale for BTX-A injections in TOS is that weakening the muscles that specifically impinge upon the brachial plexus trunks and cords may lead to symptom reduction [3]. Jordan *et al.* [39] injected 100 units of Botox distributed throughout the anterior and middle scalene muscles and trapezius. This study used electrophysiologically and fluoroscopically guided selective injection of the scalene muscles with BTX-A. Sixty four percent of the 22 patients had greater than 50% reduction of symptoms. The results had a mean duration of symptom relief of 88 days. Two patients in the study experienced symptoms of mild subjective dysphagia. No other complications were found. These results support the hypothesis that BTX-A can be helpful in alleviating symptoms of TOS [39]. BTX-A may help in the prediction of individuals who may benefit from scalenectomy as a surgical option [38]. The role of BTX-A as a predictor of a good surgical outcome is supported by a previous study that demonstrated that there was a 94% surgical success rate in those patients that had previously shown temporary improvement with anesthetic and steroid injection into the scalene muscles [51]. 

A recent study by Torrianni *et al.* [3] of 41 individuals diagnosed with NTOS who underwent ultrasound guided BTX-A injections into anterior scalene, pectoralis minor and subclavius muscles showed encouraging results. Twelve units of BTX-A were injected to the scalene muscle and subclavius muscle and fifteen units were injected to the pectoralis minor muscle. Symptom improvement as measured on a visual analogue scale (VAS) for pain revealed that 69% had significant improvement, with a mean reduction in the VAS after the procedure of 4 cm. The mean duration of symptom improvement was 31 days. There were no complications in this study [3].

Danielson *et al.* [38] demonstrated that BTX-A injections under ultrasound guidance could be used to treat arterial TOS. In this case report, they injected a 28 year old male with subclavian artery compression on Doppler ultrasound, with 15 units of BTX-A into his anterior scalene muscle. Three weeks following injection the patient was found to have a clinical improvement in subclavian artery blood flow as demonstrated by Doppler ultrasound. The increase in subclavian artery flow rate just distal to the area of stenosis increased from 87.7 cm/s (prior to injection) to 119.1 cm/s (3 weeks post-injection). It is important to note that arterial TOS was defined only by positional compression of the subclavian artery measured on Duplex ultrasound. It is possible that the patient had symptoms that some would have attributed to brachial plexus compression and not arterial insufficiency. The beneficial effects of anterior scalene muscle injection with BTX-A in this case could possibly be attributable to diminished compression and irritation of the adjacent brachial plexus nerves, and the improvement in the Duplex-derived subclavian artery flow rate observed 3 weeks later may not have had a relationship to the reduction in symptoms. However, this case report suggests that BTX-A has potential in the treatment of TOS and possibly in the prediction of individuals who may benefit from scalenectomy as a surgical option [38]. 

A recent prospective longitudinal study by Christo *et al.* [37] examined the effectiveness of injecting 20 units of BTX-A into the anterior scalene muscles under CT guidance in the treatment of NTOS in 27 patients who had failed physical therapy. The outcome measure was pain as measured by the Short-form McGill Pain Questionnaire before the BTX-A injection and at one month, two and three months post injection. They found a decline in pain three months following BTX-A injection into the anterior scalene muscle that was statistically significant in months one and two post injection. The authors concluded that BTX-A injection into the anterior scalene muscle may offer an effective and minimally invasive treatment for NTOS [37]. Some clinicians would caution that a 3-month improvement in symptoms does not really suffice to establish an effective treatment for what can sometimes become a chronic and disabling condition. Others have suggested that this should be interpreted as an approach that may postpone surgical treatment that would otherwise have been undertaken earlier. 

Unfortunately, none of the reports described above have evaluated the use of BTX-A in the management of TOS in a randomized controlled trial (RCT). At our institution, an investigation of the effect of BTX-A injections into the anterior and middle scalene muscles on pain in individuals with NTOS was recently completed [21]. This was a double blind, randomized, parallel group trial. Thirty eight subjects with NTOS were randomized into two groups. One group received BTX-A injections and the other group received normal saline injections. All injections were done under electrophysiologic guidance and were divided equally between the anterior and middle scalene muscles. Those in the treatment group received a total of 75 units of BTX-A reconstituted with normal saline and those in the placebo group received normal saline alone. Follow up of patients was at 6 weeks, 3 months and 6 months. Pain on a horizontal VAS was the primary outcome measure. Secondary outcome measures were paraesthesias measured on a VAS, and function measured using the Disabilities of the Arm, Shoulder and Hand (DASH) and SF-36 questionnaires. Interestingly, this study found there were no clinically or statistically significant improvements in subjects’ symptoms or function following BTX-A injections compared with placebo. There are several factors that may have contributed to this finding. For example, the mean duration of symptoms in the treatment group was six years, compared to three years in the placebo group. It is probable that some of the subjects had developed chronic pain with centralization such that one treatment modality in isolation was inadequate for these patients. It is possible that the dose of BTX-A may not be optimum as, in practice, many clinicians use a total dose of 100 units. However, given that other studies have shown positive results with considerably lower doses [3,37,38,39] this requires further study. In this study, the mean baseline pain score was quite low at 5 mm in the treatment group and 14 mm in the placebo group. It is possible these levels were too low to allow detection of a significant change in pain scores. Future studies with a baseline pain score of at least 40 mm as an inclusion criteria should be considered. It would be interesting to know if any of these subjects were considered candidates for surgical treatment with or without the study option or how many of these patients eventually underwent surgical treatment for NTOS.

## 10. Summary

TOS can be divided into three types, arterial, venous and neurogenic. The majority of TOS is neurogenic. It can be difficult to diagnose because there is no standardized objective test that can be used and the clinician must rely on history and several positive findings on physical exam. New research suggests that medial antebrachial cutaneous nerve conduction is a sensitive way to detect pathology in the lower trunks of the brachial plexus which is promising for future research. Treatment options continue to be conservative and surgical. However, for those who have failed physical therapy there is research to suggest that BTX may help with symptom relief. There is a growing body of evidence that BTX has a role in the management of pain and in the treatment of TOS. The decision to treat a patient with BTX should be undertaken carefully as there are potential complications to this injection, such as muscle weakness, dysphagia and dysphonia. As well, many patients would require repeat injections as the effect of the BTX wears off and this may put them at an increased risk for the development of antibodies to the BTX and make subsequent treatments less effective. However, in the appropriate patient population this treatment may possibly help those with NTOS avoid or postpone surgery or act as an outcome predictor for surgical intervention in the future. However, given that there has been conflicting evidence, further research is required using randomized controlled trials. Future research should focus on the optimal treatment dose of botulinum toxin injections, choice of muscles for injection and patient factors such as baseline pain scores and length of time the individual has had symptoms. BTX injection for the treatment of TOS is an exciting area of new and upcoming research that will no doubt add important information to the growing literature in this field. 

## References

[1] Sanders R.J., Hammond S.L., Rao N.M. (2007). Diagnosis of thoracic outlet syndrome. J. Vasc. Surg..

[2] Sanders R.J., Hammond S.L., Rao N.M. (2008). Thoracic outlet syndrome: A review. Neurologist.

[3] Torriani M., Gupta R., Donahue D. (2010). Botulinum toxin injection in neurogenic thoracic outlet syndrome: Results and experience using an ultrasound-guided approach. Skeletal Radiol..

[4] Cormier J.M., Amrane M., Ward A. (1989). Arterial complications of the thoracic outlet syndrome: Fifty five operative cases. J. Vasc. Surg..

[5] Ferrante M.A. (2012). The thoracic outlet syndromes. Muscle Nerve.

[6] Schneider D.B., Dimuizio P.J., Martin N.D. (2004). Combination treatment of venous thoracic outlet syndrome: Open surgical decompression and intraoperative angioplasty. J. Vasc. Surg..

[7] Jordan S.E., Machleder H.I. (1998). Diagnosis of thoracic outlet syndrome using electrophysiologically guided anterior scalene blocks. Ann. Vasc. Surg..

[8] Kai Y., Oyama M., Kurose S., Inadome T., Oketani Y., Masuda Y. (2001). Neurogenic thoracic outlet syndrome in whiplash injury. J. Spin. Dis..

[9] Magee D.J. (2008). Orthopedic Physical Assessment.

[10] Kleinrensink G., Stoeckart R., Mulder P., Hoek G., Broek T., Vleeming A., Snijders C. (2001). Upper limb tension tests as tools in the diagnosis of nerve and plexus lesions. Anatomical and biochemical aspects. Clin. Biomech..

[11] Selvaratnam P., Glasgow E.F., Mathias T., Dalzeill B.A., Snowsill J.C. (1987). The Discriminative Validity of the Brachia Plexus Tension Test. Proceedings of the Fifth Biennial Conference.

[12] Quinter J.L. (1989). Clinical practice study of upper limb pain and paraesthesiae following neck injury in motor vehicle accidents: Assessment of the brachial plexus tension test of elvey. Br. J. Rhem..

[13] Elvey R.L., Glasgow E.F., Twomey L. (1979). The Pathoanatomical Origin of Arm Pain. Aspects of Manipulative Therapy.

[14] Roos D.B. (1966). Transaxillary approach for first rib resection to relieve thoracic outlet syndrome. Ann. Surg..

[15] Adson A.W. (1947). Surgical treatment for symptoms produced by cervical ribs and the scalenus anticus muscle. Surg. Gynecol. Obstet..

[16] Passero S., Paradiso C., Giannini F. (1994). Diagnosis of thoracic outlet syndrome. Relative value of electrophysiological studies. Acta Neurol. Scand..

[17] Aminoff M.J., Olney R.K., Parry G.J. (1988). Relative utility of different electrophysiologic techniques in the evaluation of brachial plexopathies. Neurology.

[18] Tolson T.D. (2004). EMG for thoracic outlet syndrome. Hand Clinic..

[19] Nishida T., Price S.J., Minieka M.M. (1993). Medial antebrachial cutaneous nerve conduction in true neurogenic thoracic outlet syndrome. Electromyogr. Clin. Neurophysiol..

[20] Kothari M.J., Macintosh K., Heistand M. (1998). Medial antebrachial cutaneous sensory studies in the evaluation of neurogenic thoracic outlet syndrome. Muscle Nerve.

[21] Finlayson H.C., O’Connor R.J., Brasher P.M., Travlos A. (2011). Botulinum toxin injection for the management of thoracic outlet syndrome: A double blind, randomized controlled trial. Pain.

[22] Sanders R.J., Jackson C.G.R., Banchero N. (1990). Scalene muscle abnormalities in traumatic thoracic outlet syndrome. Am. J. Surg..

[23] Ellison D.W., Wood V.E. (1994). Trauma related thoracic outlet syndrome. J. Hand Surg..

[24] Sallstrom J., Schmidt H. (1984). Cervicobrachial disorders in certain occupations with special reference to compression in the thoracic outlet. Am. J. Ind. Med..

[25] Hagberg M., Wegman D. (1987). Prevalence rates and odds ratios of shoulder neck diseases in different occupational groups. Br. J. Ind. Med..

[26] Mandel S. (1987). Neurologic syndromes from repetitive trauma at work. Postgrad. Med..

[27] Sanders R.J., Hammond S.L. (2002). Management of cervical ribs and anomalous first ribs causing neurogenic thoracic outlet syndrome. J. Vasc. Surg..

[28] Sanders R.J., Pearce W.H. (1989). The treatment of thoracic outlet syndrome: A comparison of different operations. J. Vasc. Surg..

[29] Sanders R.J., Rao N.M. (2010). The forgotten pectoralis minor syndrome: 100 operations for pec minor syndrome alone or accompanied by neurogenic thoracic outlet syndrome. Ann. Vasc. Surg..

[30] Piyawinijwong S., Sirisathira N. (2010). Supernumerary subclavius muscle in thais: Predisposing cause of thoracic outlet syndrome. J. Med. Assoc. Thai..

[31] Forcada P., Rodriguez-Niedenfuhr M., Llusa M., Carrera A. (2001). Subclavius posticus muscle: Supernumerary muscle as a potential cause for thoracic outlet syndrome. Clin. Anat..

[32] Haven H. (1939). Neurocirculatory scalenus anticus syndrome in the presence of developmental defects in the first rib. Yale J. Biol. Med..

[33] Crosby C.A., Webbe M.A. (2004). Conservative treatment of thoracic outlet syndrome. Hand Clin..

[34] Buchanan P.A., Ultrich B.D. (2003). The Feldenkrais method: A dynamic approach to changing motor behaviour. Res. Q. Exerc. Sport.

[35] Voerman G.E., Vollenbroek-Hutton M.M., Hermens H.J. (2006). Changes in pain, disability and muscle activation patterns in chronic whiplash patients after ambulant myofeedback training. Clin. J. Pain.

[36] Braun R.M., Sahadevan D.C., Feinstein J. (2006). Confirmatory needle placement technique for scalene muscle block in the diagnosis of thoracic outlet syndrome. Tech. Hand Up. Extrem. Surg..

[37] Christo P.J., Christo D.K., Carinci A.J., Freischlag J.A. (2010). Single CT-guided chemodenervation of the anterior scalene muscle with botulinum toxin for neurogenic thoracic outlet syndrome. Pain Med..

[38] Danielson K., Odderson I.R. (2008). Botulinum toxin type A improves blood flow in vascular thoracic outlet syndrome. Am. J. Phys. Med. Rehabil..

[39] Jordan S.E., Ahn S.S., Freischlag J.A., Gelabert H.A., Machleder H.I. (2000). Selective botulinum toxin chemodenervation of the scalene muscles for treatment of neurogenic thoracic outlet syndrome. Ann. Vasc. Surg..

[40] Le E.N., Freischlag J.A., Christo P.F., Chhabra S., Wigley F.M. (2010). Thoracic outlet syndrome secondary to scleroderma treated with botulinum toxin injection. Arthritis Care Res..

[41] Ranoux D., Attal N., Morain F., Bouhassira D. (2008). Botulinum toxin type A induces direct analgesic effects in chronic neuropathic pain. Ann. Neurol..

[42] Aoki K.R. (2005). Review of proposed mechanism for the antinoceptive action of botulism toxin type A. Neurotoxicology.

[43] Mense S. (2004). Neurobiological basis for the use of botulinum toxin in pain therapy. J. Neurol..

[44] Simpson L.L. (2004). Identification of the major steps in botulinum toxin action. Annu. Rev. Pharmacol. Toxicol..

[45] Yuan R.Y., Sheu J.J., Yu J.M. (2009). Botulinum toxin for diabetic neuropathic pain: A randomized double blind crossover trial. Neurology.

[46] Carroll I., Clark J.D., Mackey S. (2009). Sympathetic block with botulinum toxin to treat complex regional pain syndrome. Ann. Neurol..

[47] Gobel H., Heinze A., Reichel G. (2006). Efficacy and safety of a single botulinum type A toxin complex treatment for the relief of upper back myfascial pain syndrome: Results from a randomized double-blind placebo controlled multicentre study. Pain.

[48] Foster L., Clapp L., Erickson M., Jabbari B. (2001). Botulinum toxin A and chronic low back pain: A randomized, double-blind study. Neurology.

[49] Singh J.A., Mahowald M.L., Noorbaloochi S. (2009). Intraarticular botulinum toxin type A for refractory shoulder pain: A randomized, double-blinded, placebo-controlled trial. Transl. Res..

[50] Qerama E., Fugslang-Frederiksen A., Jensen T.S. (2010). The role of botulinum toxin in management of pain: An evidence-based review. Curr. Opin. Anaesthesiol..

[51] Jordan S.E., Ahn S.S., Gelabert H.A. (2007). Combining ultrasonography and electromyography for botulinum chemodenervation treatment of thoracic outlet syndrome: Comparison with fluoroscopy and electromyography guidance. Pain Physician.

